# Supplementary material for: A distinctive family of L,D-transpeptidases catalyzing L-Ala-mDAP crosslinks in Alpha- and Betaproteobacteria
Source: Nat Commun. 2024 Feb 13;15:1343. doi: 10.1038/s41467-024-45620-5 (PMC10864386; doi:10.1038/s41467-024-45620-5)
Supplement: Supplementary file 1 — Supplementary Information [file 41467_2024_45620_MOESM1_ESM.pdf]

SUPPLEMENTARY INFORMATION

A distinctive family of L,D-transpeptidases catalyzing L-Ala-mDAP crosslinks in Alpha- and Betaproteobacteria

Akbar Espaillat[1,6#], Laura Alvarez[1#], Gabriel Torrens[1#], Josy ter Beek[2,3], Vega Miguel-Ruano[4], Oihane Irazoki[1], Federico Gago[5], Juan A. Hermoso[4], Ronnie Per-Arne Berntsson[2,3] and Felipe Cava[1]*

[1]Department of Molecular Biology and Laboratory for Molecular Infection Medicine Sweden, Umeå Centre for Microbial Research, SciLifeLab, Umeå University, Umeå, Sweden.

[2]Department of Medical Biochemistry and Biophysics, Umeå University, Umeå, Sweden

[3]Wallenberg Centre for Molecular Medicine, Umeå University, Umeå, Sweden

[4]Department of Crystallography and Structural Biology, Institute of Physical Chemistry "Blas Cabrera", CSIC, Madrid, Spain.

[5]Department of Biomedical Sciences & IQM-CSIC Associate Unit, School of Medicine and Health Sciences, University of Alcalá, E-28805 Madrid, Alcalá de Henares, Spain.

[6] Present address: Chr. Hansen A/S, Microbial Physiology, R&D, 2970 Hoersholm, Denmark.

[#] These authors contributed equally: Akbar Espaillat, Laura Alvarez and Gabriel Torrens.

* for correspondence: felipe.cava@umu.se

Running title: Distinctive L,D-transpeptidases in Alpha- and Betaproteobacteria.

Keywords: Peptidoglycan, L,D-transpeptidase, crosslink, structure, *Gluconobacter oxydans*.

SUPPLEMENTARY FIGURES

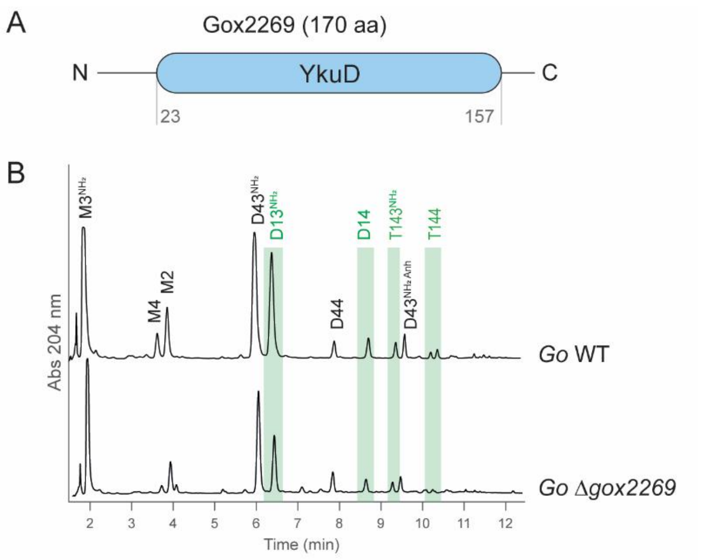

Supplementary Figure 1. Candidate YkuD-domain containing enzyme Gox2269. A) Domain structure of the protein. B) UV muropeptide profile of *G. oxydans* (*Go*) wild-type (WT) and Δ*gox2269* mutant. LD1,3-crosslinked muropeptides are highlighted in green.

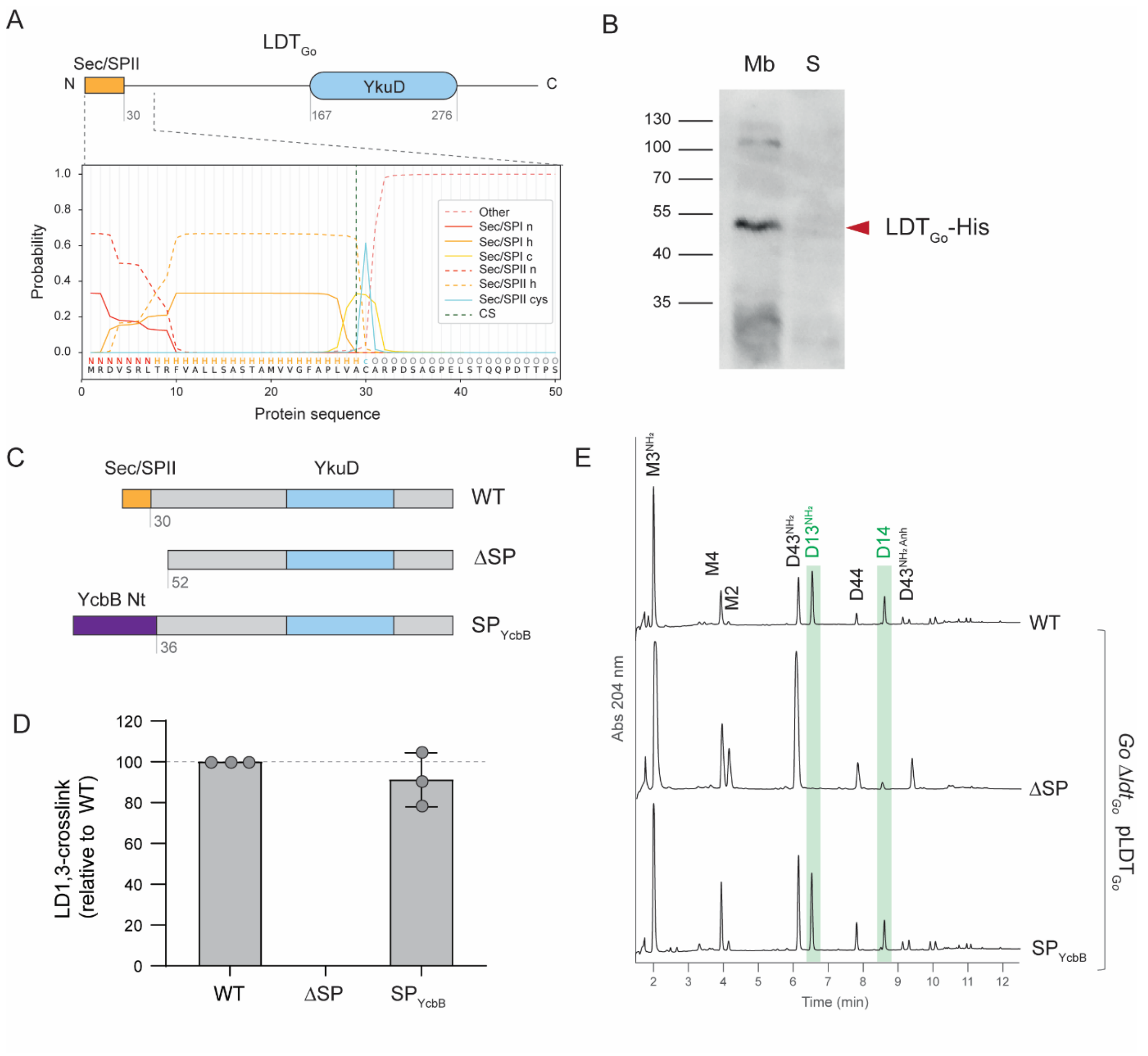

Supplementary Figure 2. LDT$_{Go}$ signal peptide. A) LDT$_{Go}$ is predicted to have an SPII lipobox (Sec/SPII). B) Detection of C-terminal His-tagged LDT$_{Go}$ in the particulate (Mb; membrane) or soluble fraction (S; cytoplasm and periplasm). Protein amount is normalized by total protein amount. C) Scheme of the LDT$_{Go}$ WT and derivatives used in panel D: one lacking its signal peptide (ΔSP) and the second replacing it by YcbB$_{Ec}$ signal peptide. Amino acid positions within LDT$_{Go}$ protein sequence are indicated. D) LD1,3-crosslinking quantifications and E) UV muropeptide profiles of *G. oxydans* Δ*ldt*$_{Go}$ strain complemented with LDT$_{Go}$ WT and derivatives indicated in panel C. Error bars in graph D represent standard deviation from mean. Source data for B and D are provided as a Source Data file.

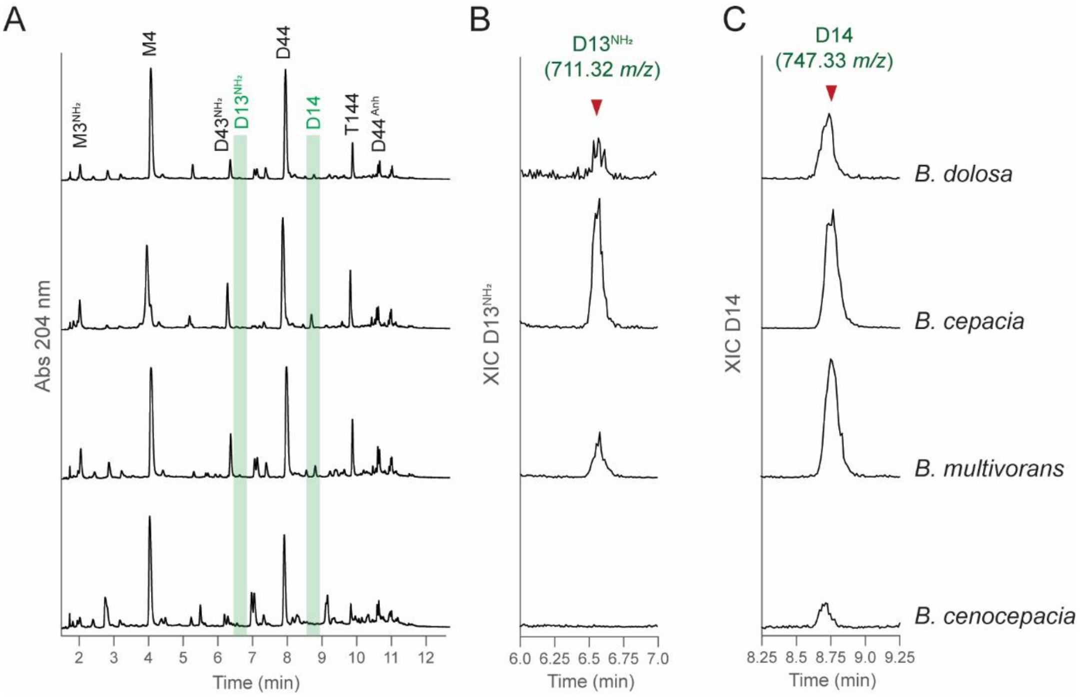

Supplementary Figure 3. LD1,3-crosslinking activity in *Burkholderia*. A) UV muropeptide profiles of the indicated *Burkholderia* species. LD1,3-crosslinked dimers $D13^{NH2}$ and D14 are highlighted in green. B, C) MS extracted ion chromatogram (XIC) traces of the indicated muropeptides.

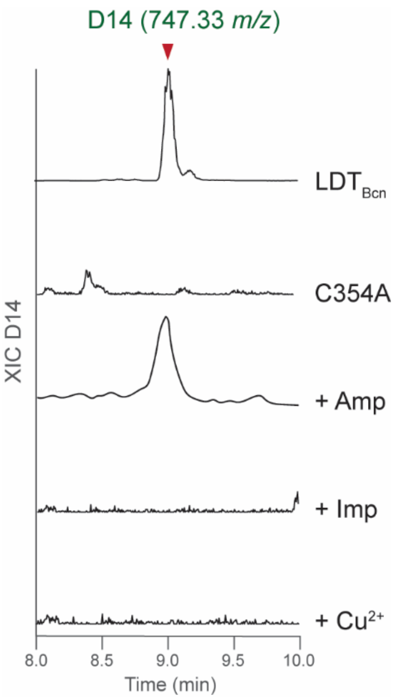

Supplementary Figure 4. Detection of LD1,3-crosslinked muropeptides *in vitro*. MS extracted ion chromatogram (XIC) trace of the D14 muropeptide in the *in vitro* activity assays of LDT_{Bcn} on M4-rich peptidoglycan sacculi (from *V. cholerae*), LDT_{Bcn} C354A point mutant (negative control) and assays with added Ampicillin 100 µg/ml (Amp), Imipenem 100 µg/ml (Imp) and copper 1 mM (Cu^{2+}).

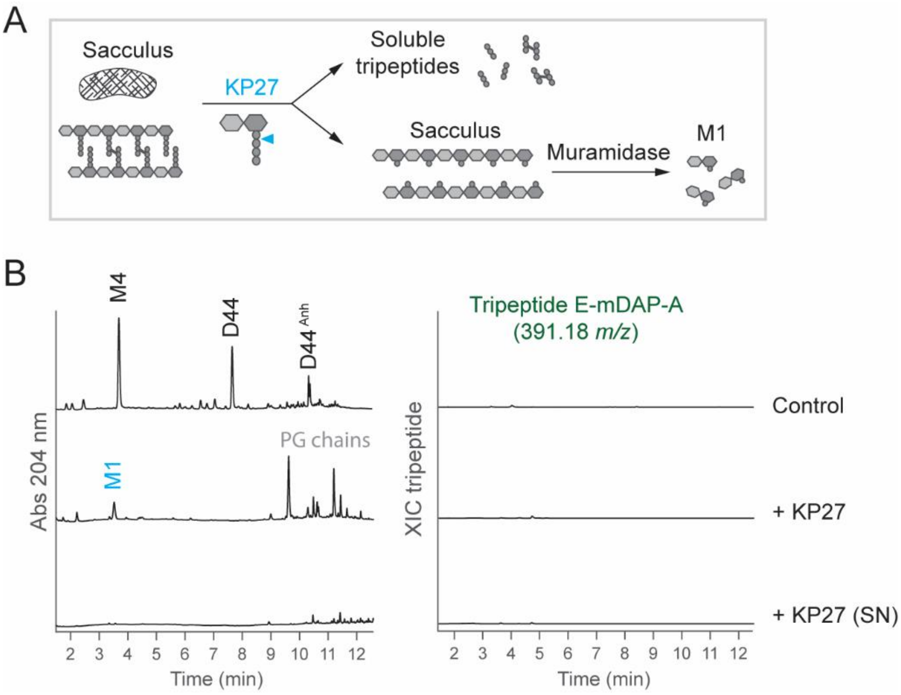

Supplementary Figure 5. Detection of tripeptide *in vitro*. A) Scheme of KP27 endopeptidase reaction on peptidoglycan sacculi. KP27 cleaving site between L-Ala[1] and D-Glu[2] is indicated with a blue arrowhead. B) UV muropeptide profiles of sacculi incubated (KP27) or not (control) with KP27 prior digestion with mutanolysin to release individual muropeptides. KP27(SN) corresponds to the analysis of free peptidoglycan soluble fragments released to the supernatant after KP27 digestion, which mostly include linear peptidoglycan chains of M1, and free tripeptides as illustrated in A. MS extracted ion chromatogram (XIC) traces of the liberated tripeptide are shown.

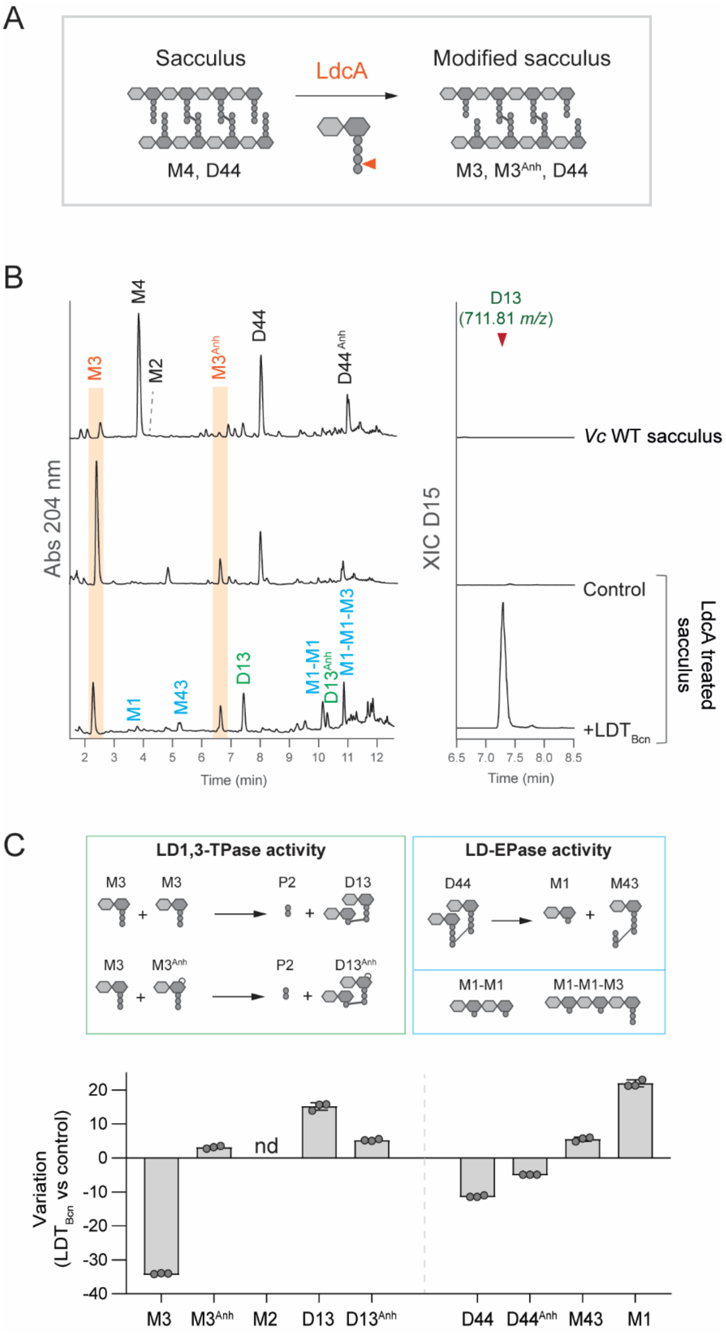

Supplementary Figure 6. LDT$_{Bcn}$ activity on peptidoglycan tripeptides. A) Scheme of the preparation of M3-enriched peptidoglycan sacculi using the LD-CPase activity of LdcA of *E. coli*. B) UV muropeptide profiles, D13 MS extracted ion chromatogram (XIC) trace and C) quantification of the relevant muropeptides indicated. Variation is calculated as difference in relative molar abundance of the muropeptide in the LDT$_{Bcn}$ vs control in the *in vitro* assays. Error bars in graph C represent standard deviation from mean. Source data for C are provided as a Source Data file.

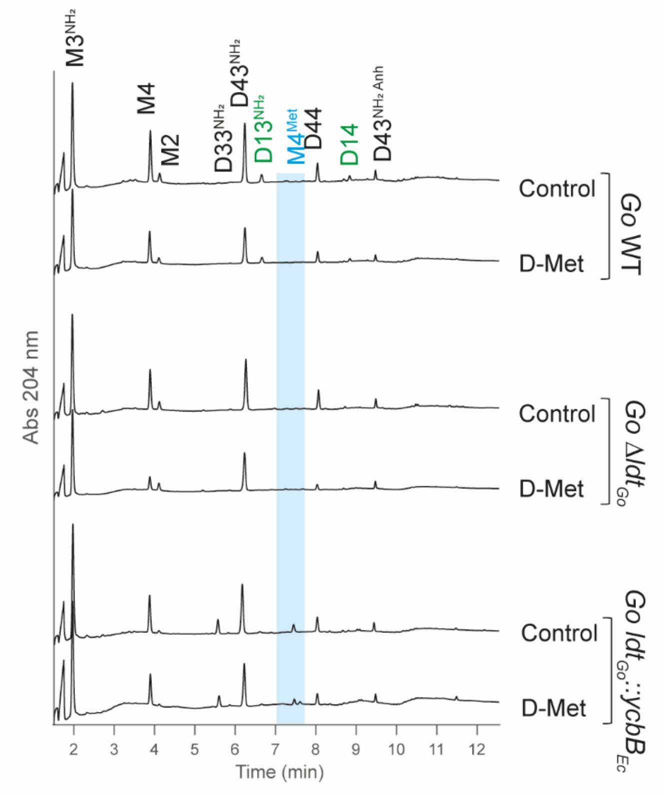

Supplementary Figure 7. Incorporation of D-Met into the peptidoglycan of *G. oxydans*. UV muropeptide profiles from cultures of *G. oxydans* (*Go*) wild-type (WT), Δldt_{Go} and a derivative strain in which the ldt_{Go} allele is replaced by $ycbB_{Ec.}$ ($ldt_{Go}::ycbB_{Ec}$) supplemented or not (Control) with 10 mM of D-Met. LD1,3-crosslinked muropeptides are highlighted in green and the D-Met-modified M4 ($M4^{Met}$) in blue.

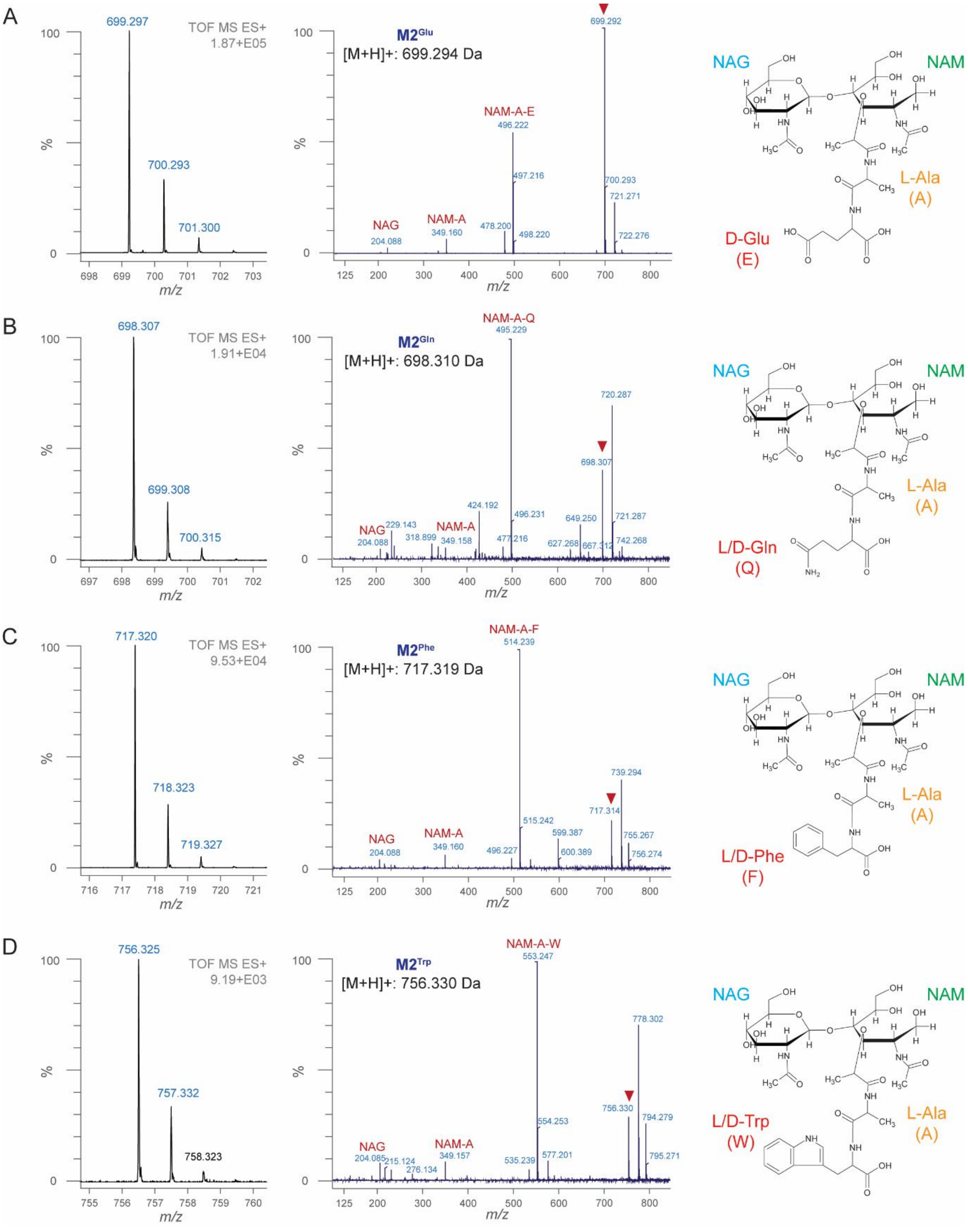

Supplementary Figure 8. Identification of M2^{X} muropeptides in *G. oxydans* peptidoglycan. Detection of the parental ion (left), its fragmentation pattern (middle) and determined structure based on the MS/MS data for A) M2^{Glu}, B) M2^{Gln}, C) M2^{Phe}, and D) M2^{Trp}.

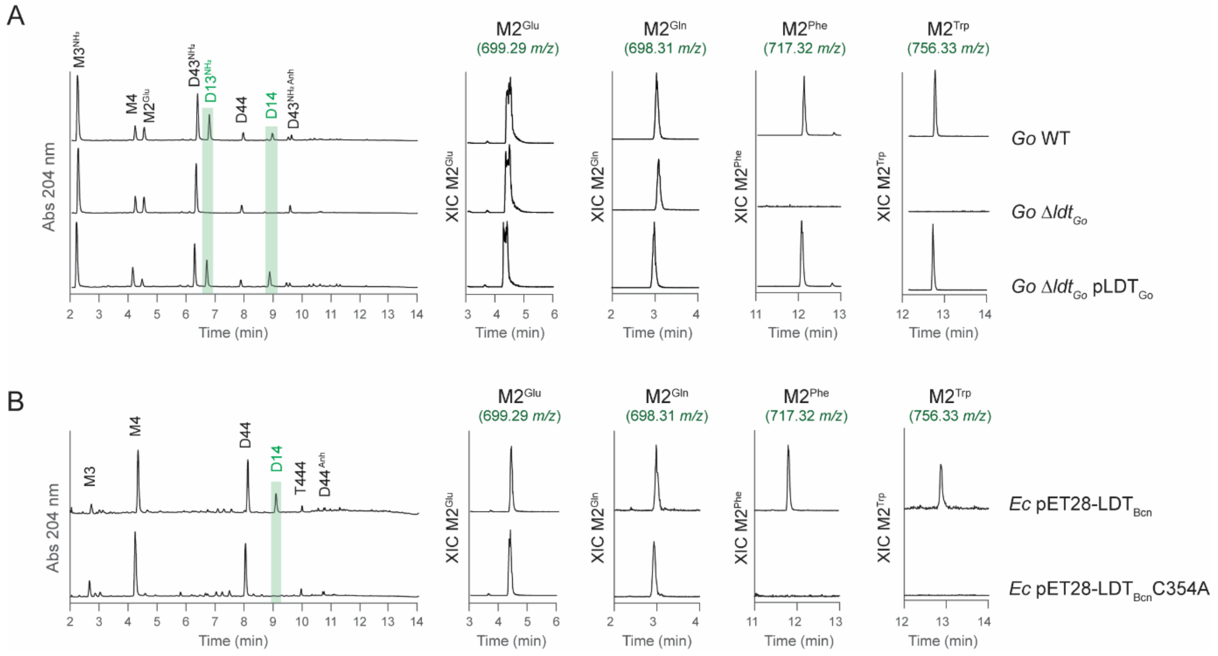

Supplementary Figure 9. Functional association between M2^X muropeptides and LD1,3-TPases. A) UV muropeptide profiles and the M2^X MS extracted ion chromatogram (XIC) traces of the indicated muropeptides in *G. oxydans* (*Go*) wild-type (WT), Δldt_{Go} mutant and complemented strains. (B) UV muropeptide profiles and the indicated M2^X MS extracted ion chromatogram (XIC) traces of the indicated muropeptides in *E. coli* BL21 expressing LDT_{Bcn} or its catalytically inactive derivative C354A.

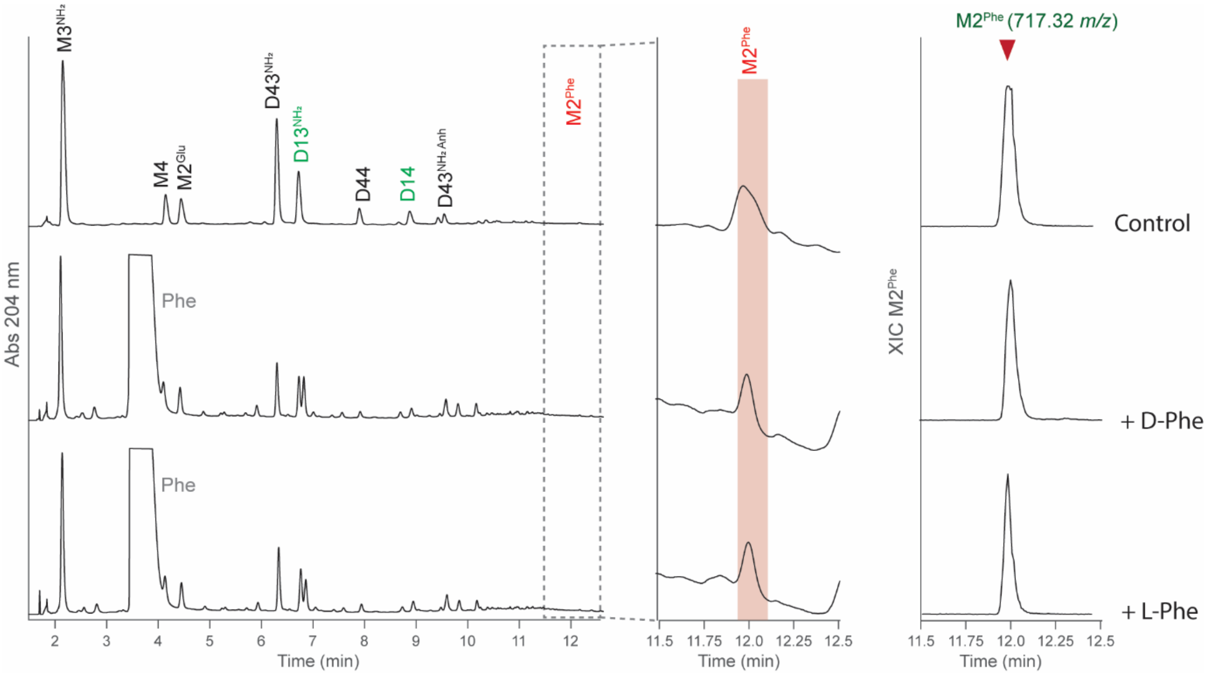

Supplementary Figure 10. Detection of M2^{Phe} *in vitro*. UV muropeptide profiles and zoom-in of *in vitro* amino acid exchange reactions using *G. oxydans* (*Go*) wild-type (WT) peptidoglycan sacculi as substrate +/- 10 mM of D- or L-Phe, incubated with LDT$_{Bcn}$. The M2^{Phe} MS extracted ion chromatogram (XIC) traces are indicated.

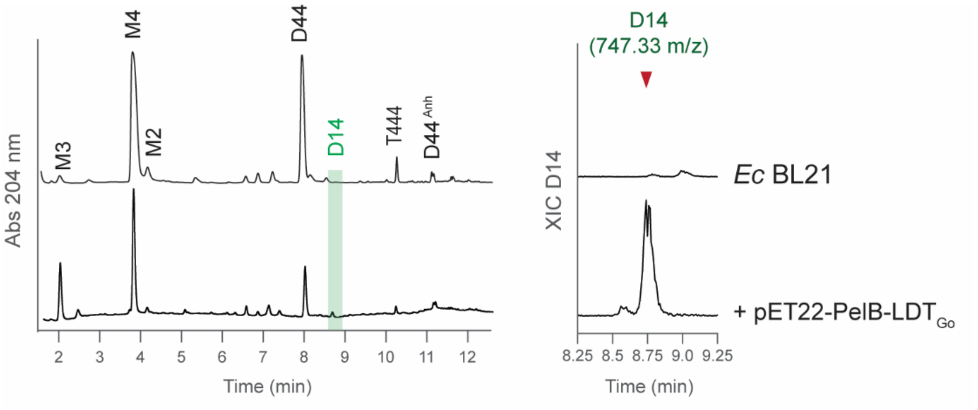

Supplementary Figure 11. *In vitro* activity of crystallized LDT$_{Go}$. Muropeptide profile of *E. coli* (*Ec*) BL21 expressing PelB-LDT$_{Go}$ and extracted ion of the D14 muropeptide. The N-terminal PelB leader sequence from the pET22b(+) plasmid directs the protein to the periplasmic space.

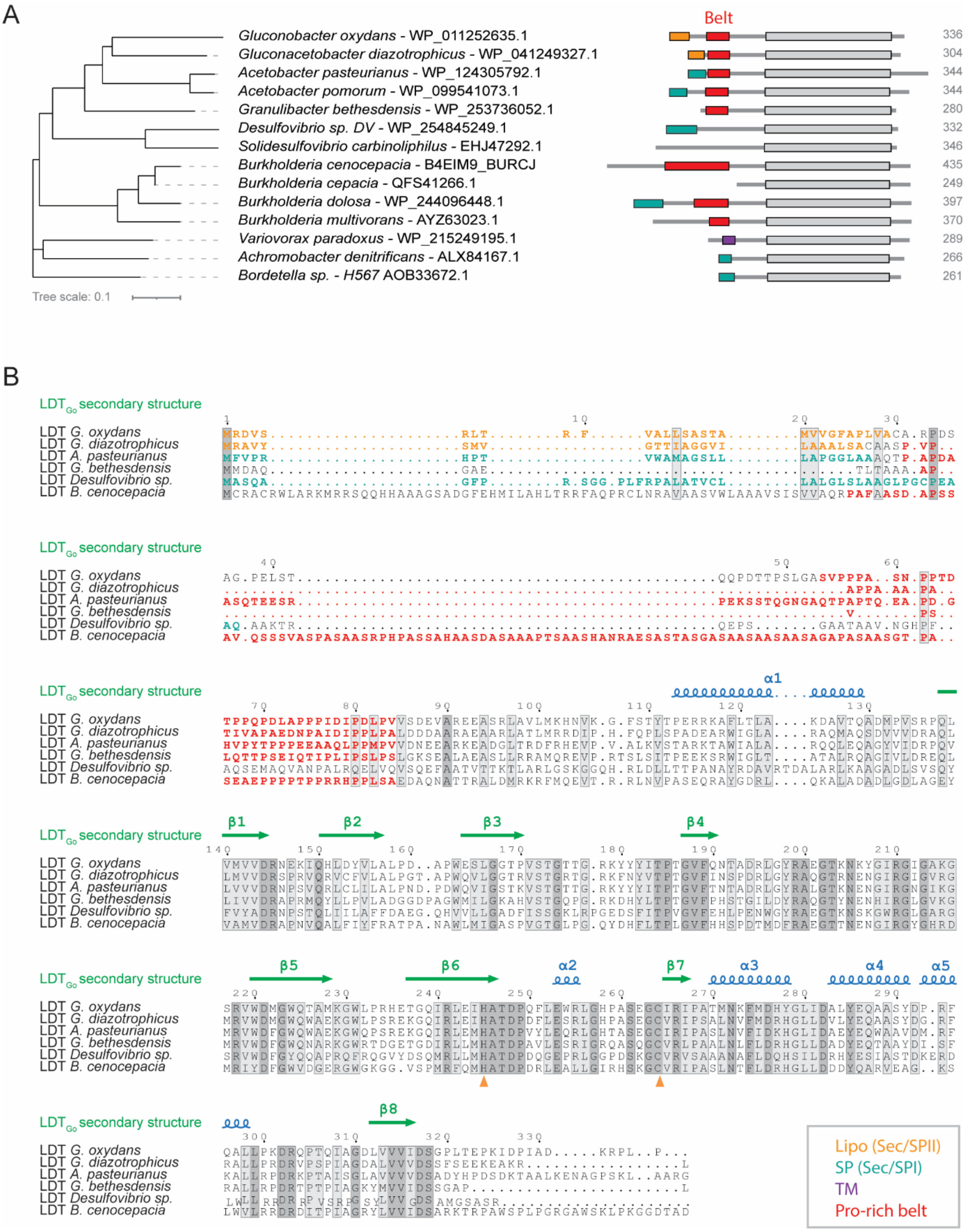

Supplementary Figure 12. Conservation of the Pro-rich belt. A) Domain analysis of representative LDT_{Go}-like proteins from different bacterial taxa. Lipoprotein signal peptide SPII (Sec/SPII) is indicated in orange, translocation signal peptide (SPI/SPI) in blue,

transmembrane domain ™ in purple, the Pro-rich belt in red, and the YkuD domain in grey. Prediction of the signal peptides was performed using SignalP 6.0. B) Multiple sequence alignment of the indicated LDT_{Go}-like proteins from different bacterial species. Secondary structure of LDT_{Go} is shown immediately above the alignment. Beta sheets and alpha helices are indicated in blue and green, respectively, and the conserved Cysteine and Histidine catalytic residues are indicated with orange arrows. Colors in the sequence alignment are as described in A.

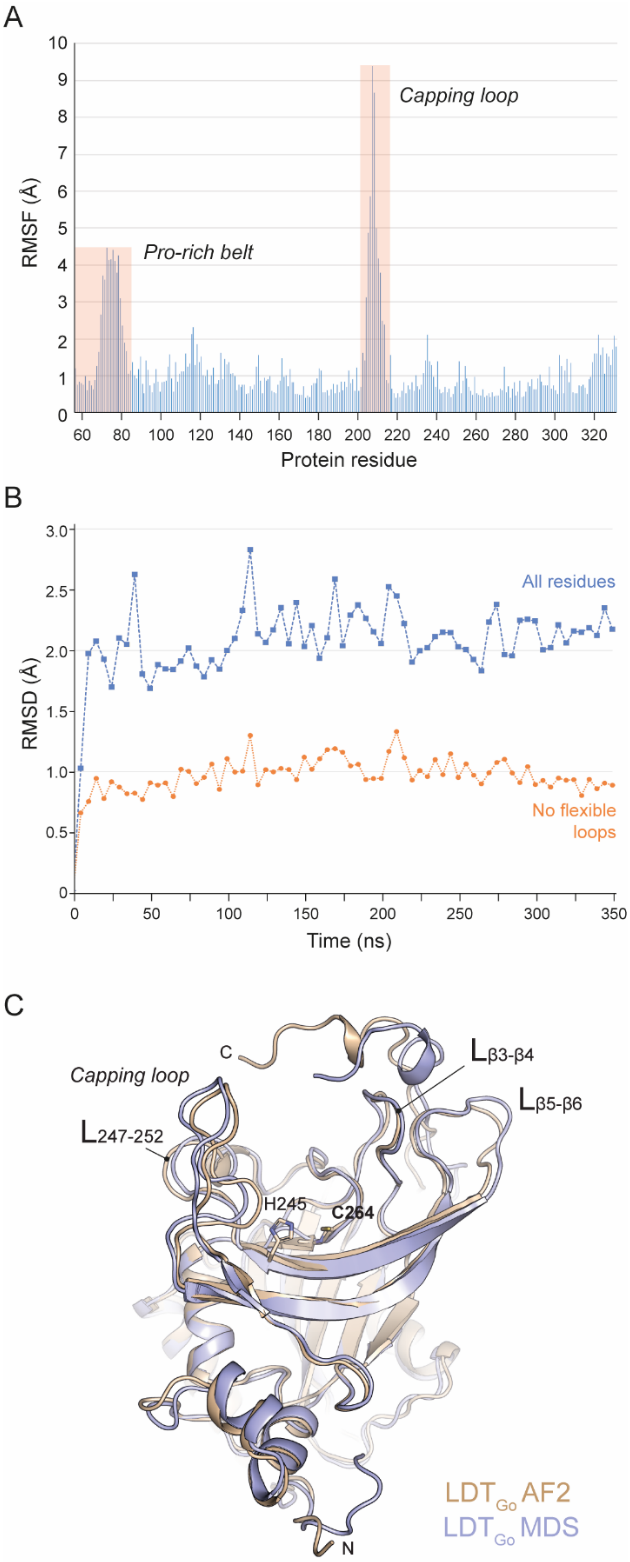

Supplementary Figure 13. Conformational variability of LDT$_{Go}$ during the molecular dynamics (MD) simulations. A) Root-mean-square fluctuation (RMSF) during the 350 ns MD simulations of the crystallographic structure. Regions experiencing larger fluctuations are indicated in the orange boxed areas and labeled. B) Evolution of the Cα trace (RMSD) over the

course of 350 ns of MD simulation with respect to the initial structure either considering all residues (blue line) or excluding the flexible loop regions highlighted in A (orange line). C) Comparison between the LDT_{Go} AlphaFold2 (AF2, light brown) model and the molecular dynamics simulation (MDS, blue) model of LDT_{Go} lacking the belt. Relevant loops and catalytic residues are indicated.

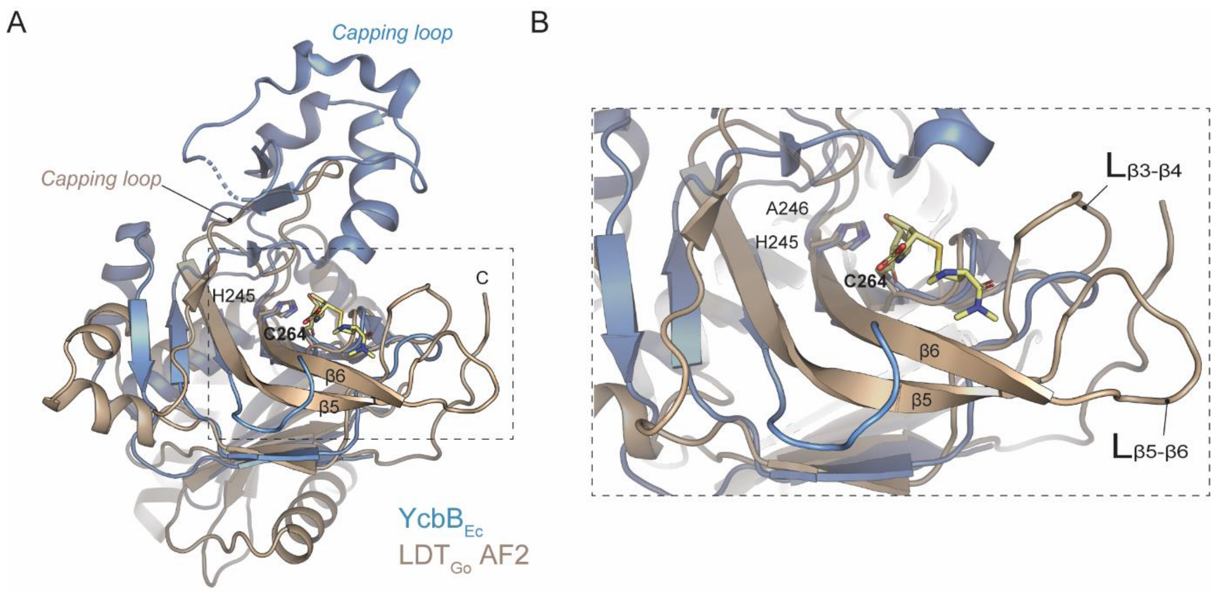

Supplementary Figure 14. Distinctive active site organization between 3,3 and 1,3 LD-TPases. A) Comparative overview between $YcbB_{Ec}$ (blue) bound to meropenem (yellow) and the Alphafold2 model of LDT_{Go}, (AF2, light brown). B) Zoom-in view of the active site. Relevant loops and catalytic residues are indicated.

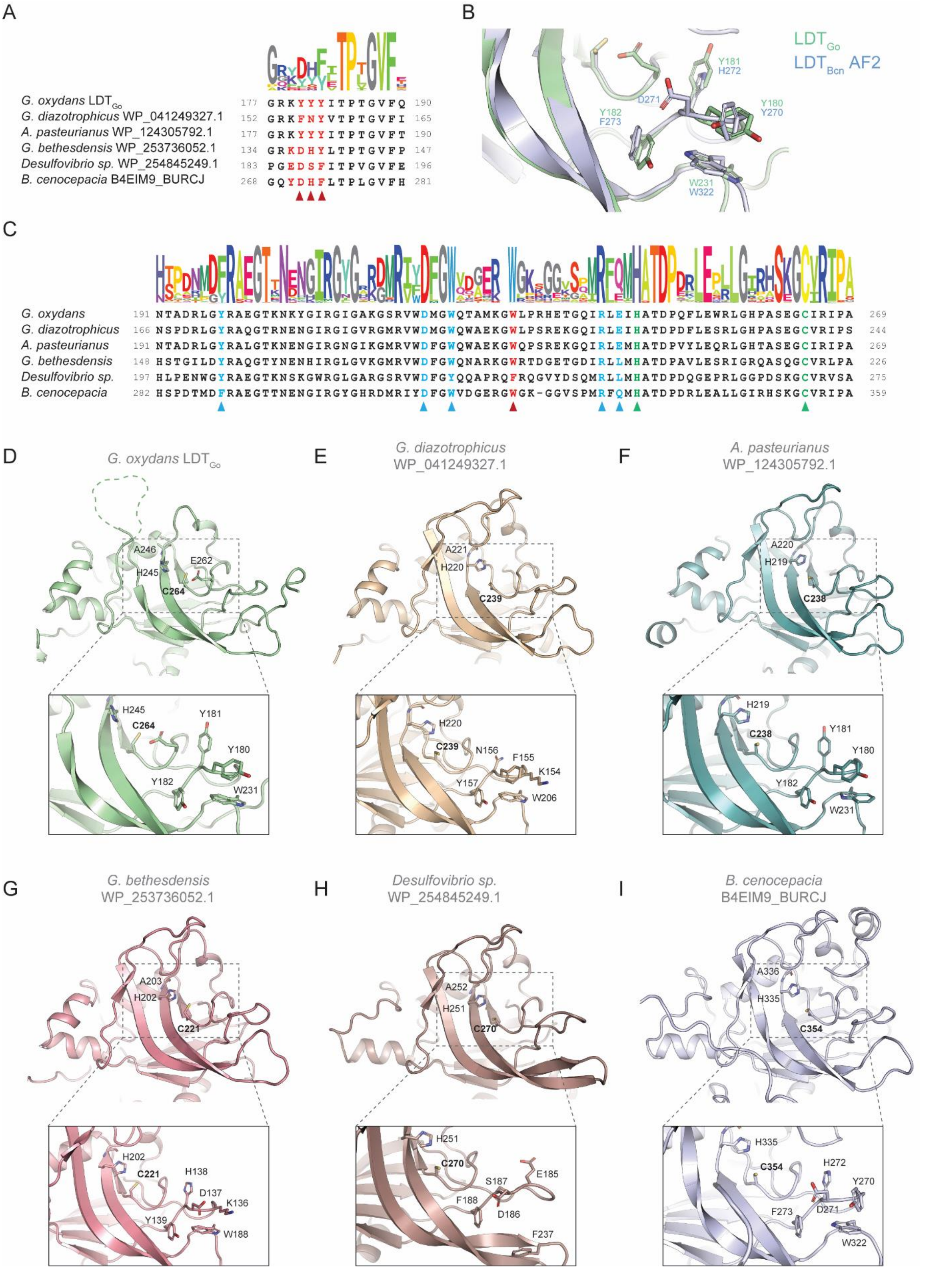

Supplementary Figure 15. Sequence and structure identity between LDT_{Go}-like proteins.

A) Conservation of the triad predicted to interact with NAM moiety at the donor site of LDT_{Go}.

B) Zoom-in view of the triad organization between the LDT_{Go} structure (green) which displays a YYY triad and the LDT_{Bcn} Alphafold2 model (AF2, blue) which displays a DHF triad. C) Comparative sequence analysis of the listed LDT_{Go} orthologs. Relevant conserved residues important substrate stabilization are indicated. D-I) Comparison of LDT_{Go} structure and Alphafold2 models of the indicated LDT_{Go} orthologs. Zoom-in view of the donor site of the active domain is shown. Relevant loops and residues are indicated.

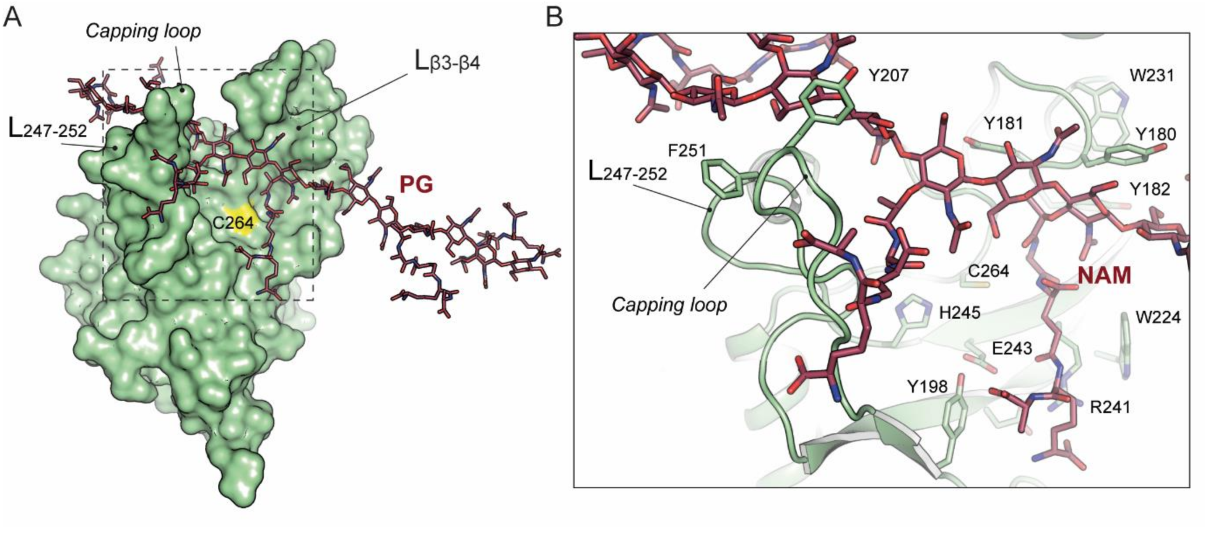

Supplementary Figure 16. Modelling of a large PG chain attached to the active site of LDT_{Go}. A) Molecular surface of LDT_{Go} (green) with the catalytic C264 highlighted in yellow. The PG layer is represented as capped sticks (C atoms colored in dark red). The positions of the relevant loops are indicated. B) Detailed view of the interaction of the PG chain in the LDT_{Go} active site. Relevant residues in the protein are represented as capped sticks and labeled. While the interactions are preserved for the NAM-peptide moiety close to the catalytic C264 residue (see Figure 6), expansion to a larger PG chain shows that the capping loop and $L_{247-252}$ also play a relevant role in stabilization of the glycan chain far from the active site by the aromatic residues Y207 (from the capping loop) and the F251 (from $L_{247-252}$).

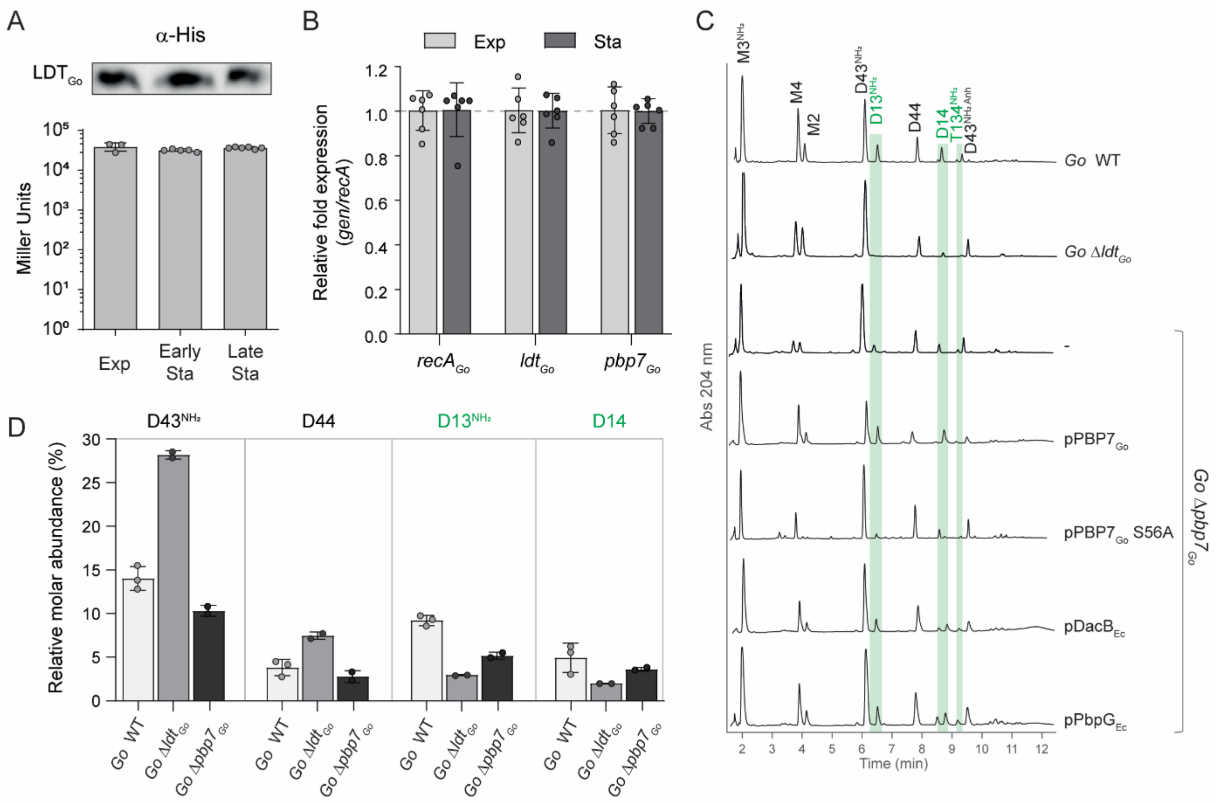

Supplementary Figure 17. PBP7$_{Go}$ controls the *in vivo* levels of LD1,3-crosslinking. A) Detection of C-terminal His-tagged LDT$_{Go}$ protein levels by Western blot and transcriptional levels by beta-galactosidase activity (Miller units) assays from cultures collected at exponential (Exp), early stationary (Early Sta) and stationary (Sta) growth phase as indicated in the methods section. Protein amount is normalized by total protein amount. B) Quantitative RT-PCR analysis of *ldt*$_{Go}$ and *pbp7*$_{Go}$ genes in exponential (Exp) and stationary (Sta) growth phases. The house keeping gene *recA*$_{Go}$ is used as control. Data from three replicas in two independent experiments was analyzed using the $2^{-\Delta\Delta Ct}$ analysis method. C) UV muropeptide profiles and quantifications of *G. oxydans* WT, Δ*ldt*$_{Go}$, Δ*pbp7*$_{Go}$ and complemented strains as indicated. D) Relative molar abundance (%) of the indicated muropeptides in *G. oxydans* WT, Δ*ldt*$_{Go}$ and Δ*pbp7*$_{Go}$ strains. Error bars in graphs A-B and D represent standard deviation from mean. Source data for A-B and D are provided as a Source Data file.

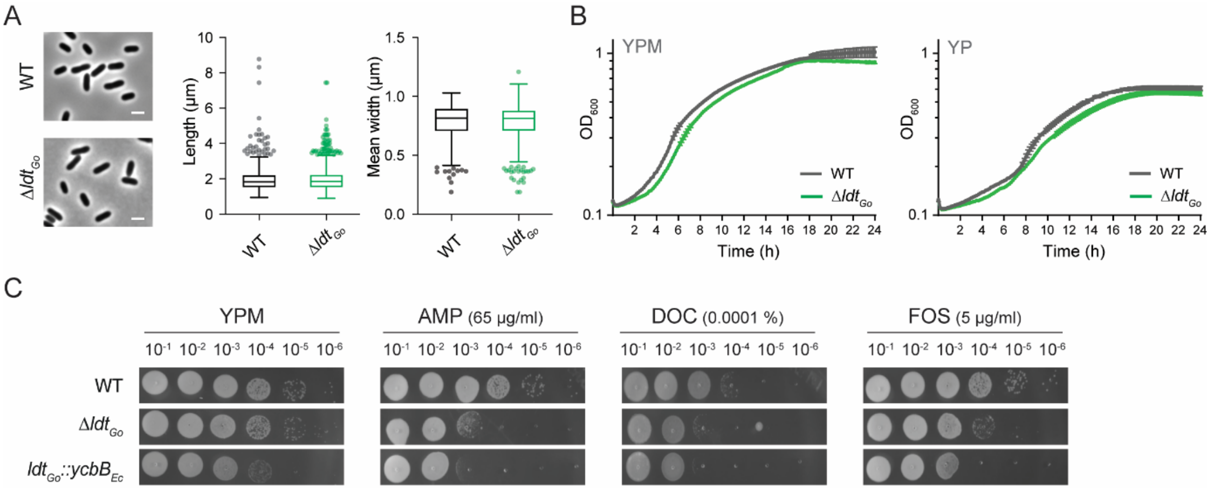

Supplementary Figure 18. Phenotypic characterization of the *G. oxydans* Δ*ldt*$_{Go}$ mutant. A) Phase contrast microscopy of wild-type (WT) and Δ*ldt*$_{Go}$ mutant. Scale bar: 2 µm. Cell length and mean width of the WT (n = 703) and Δ*ldt*$_{Go}$ mutant (n = 1587) are indicated. No significant differences found, pValue = 0.8864, applying non-parametric Mann-Whitney U test. B) Growth curves of the WT (grey) and Δ*ldt*$_{Go}$ mutant (green) grown in YPM and YP medium. C) Serial dilutions (10^{-1} to 10^{-6}) from overnight cultures of wild-type (WT), Δ*ldt*$_{Go}$ mutant and allelic exchange with YcbB$_{Ec}$ (*ldt*$_{Go}$*::ycbB*$_{Ec}$) were spotted onto YPM agar plates supplemented with Ampicillin 65 µg/ml (AMP), deoxycholate 0.0001 % (w/v) (DOC) and Fosfomycin 5 µg/ml (FOS). Growth on non-supplemented plate (YPM) was used as control. Source data for A-B are provided as a Source Data file.

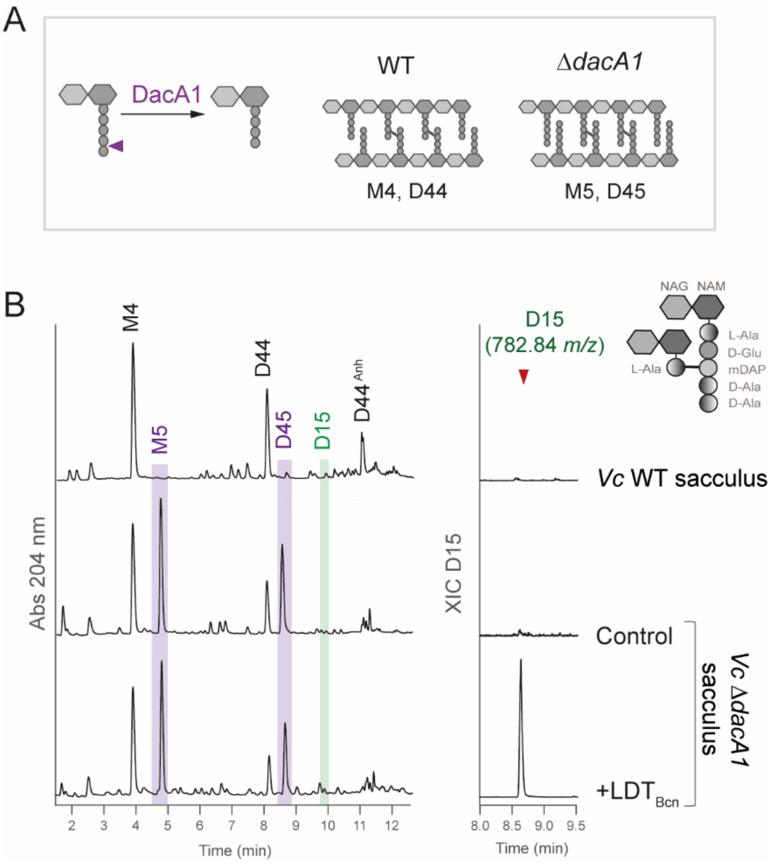

Supplementary Figure 19. LDT$_{Bcn}$ activity on peptidoglycan pentapeptides. A) Scheme of the DacA1 DD-CPase activity and the muropeptide architecture of the peptidoglycan of *V. cholerae* WT and its Δ*dacA1* mutant derivative strain (M5-enriched). B) UV muropeptide profiles of the *V. cholerae* WT sacculi (M4 rich) and the Δ*dacA* mutant (M5-enriched, pentapeptide muropeptides highlighted in purple) treated or not (Control) with LDT$_{Bcn}$. The LD1,3-crosslinked dimer D15 is highlighted in green, and its MS extracted ion chromatogram (XIC) trace is shown in the right-side panel.

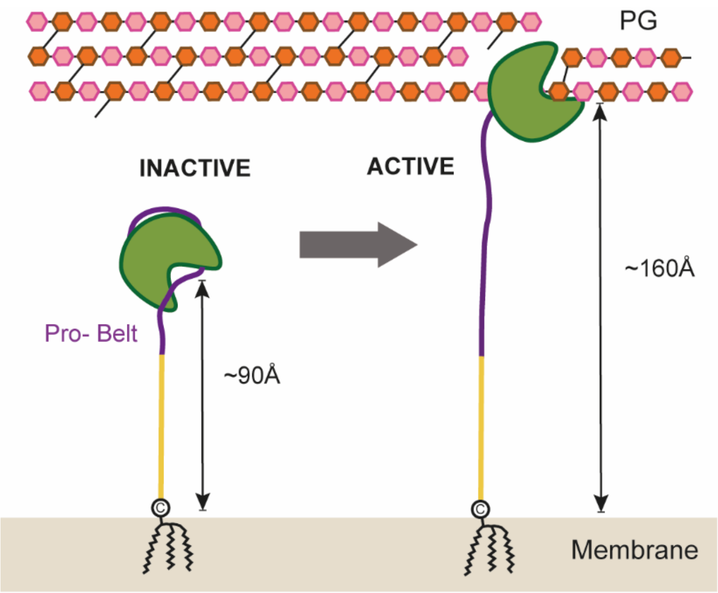

Supplementary Figure 20. Model of the regulation of LDT_{Go} by the Pro-rich belt. Graphic illustration suggesting how the detachment of the belt would facilitate both unblocking the active site and approaching it to the peptidoglycan.

SUPPLEMENTARY TABLES

Supplementary Table 1. Hydrogen bonds between the Pro-rich belt and the catalytic center.

**Pro-rich belt residue:**	**H-bond with residue:**	**Distance *:**
**Pro56 (main-chain, =O)**	Tyr181 (side-chain, OH)	2.9 Å
**Ala59 (main-chain, NH)**	Glu262 (side-chain, =O)	2.8 Å
**Ser60 (main-chain, =O)**	His245 (side-chain, NH) **	3.2 Å
**Asn61 (side-chain, =O)**	His245 (side-chain, NH) **	2.7 Å
**Asn61 (side-chain, NH_2)**	Glu262 (side-chain, O^-)	3.1 Å
**Thr64 (main-chain, =O)**	Leu196 (main-chain, NH)	3.0 Å
**Thr64 (side-chain, OH)**	Gly197 (main-chain, NH)	2.8 Å
**Thr66 (main-chain, NH)**	Asp194 (main-chain, =O)	2.9 Å
**Thr66 (side-chain, OH)**	Ala193 (main-cahin, =O)	2.9 Å
**Pro67 (main-chain, =O)**	Arg294 (side-chain, NH_2)	2.8 Å
**Pro80 (main-chain, =O)**	Gln287 (side-chain, NH_2)	2.8 Å
**Pro83 (main-chain, =O)**	Arg136 (side-chain, NH)	2.7 Å

* As measured between the oxygen and the nitrogen/oxygen.
** As both interactions are with the same nitrogen from the ring of the catalytic His245, only one of these will form at the time. Probably the interaction with Asn61 will dominate as it is over the shortest distance.

Supplementary Table 2. Strains.

FC code	Name	Description	Reference/ Source
***Gluconobacter oxydans* DSM7145 derivative strains:**			
FC1604	wild-type	Cfx^R	[1]
FC3590	Δ*gox2269* (putative *ykuD* gene)	Deletion mutant of *gox2269.*	This study
FC2150	Δ*gox1074* (Δldt_{Go})	Deletion mutant of ldt_{Go}	This study
FC3591	Δ*gox1074* pGox1074 (Δldt_{Go} $pLDT_{Go}$)	FC2150 strain complemented by expressing ldt_{Go} WT allele from the plasmid pSEVA238 (pGox1074). Kan^R.	This study
FC3592	Δ*gox1074* pGox1074 H245A	FC2150 strain complemented by expressing ldt_{Go} H245A mutant allele from the plasmid pSEVA238 (pGox1074 H245A). Kan^R.	This study
FC3593	Δ*gox1074* p*Gox1074* C264A	FC2150 strain complemented by expressing ldt_{Go} C264A mutant allele from the plasmid pSEVA238 (pGox1074 C264A). Kan^R.	This study
FC3594	Δ*ldtgo* $pLDT_{Go}$ ΔSP	FC2150 strain complemented by expressing ldt_{Go} allele lacking the first 52 amino acids from the plasmid pSEVA238 ($pLDT_{Go}$ ΔSP). Kan^R.	This study
FC3595	Δ*ldtgo* $pLDT_{Go}$ SP_{YcbB}	FC2150 strain complemented by expressing ldt_{Go} in which the signal peptide (first 35 AA) has been replaced by the SP (first 100 AA) of $YcbB_{Ec}$ amino acids from the plasmid pSEVA238 ($pLDT_{Go}$ SP_{YcbB}). Kan^R.	This study
FC3596	ldt_{Go}::$ycbB_{Ec}$	*G. oxydans* strain in which the ldt_{Go} allele has been replaced by $ycbB_{Ec}$	This study
FC3597	Δ*ldtgo* $pLDT_{Bcn}$	FC2150 strain complemented by expressing ldt_{Bcn} WT allele from the plasmid pSEVA238 ($pLDT_{Bcn}$). Kan^R.	This study
FC3598	Δ$pbp7_{Go}$	Deletion mutant of *gox0607.*	This study
FC3599	Δ$pbp7_{Go}$ $pPBP7_{Go}$	FC3598 strain complemented by expressing $pbp7_{Go}$ WT allele from the plasmid pSEVA238 ($pPBP7_{Go}$). Kan^R.	This study
FC3600	Δ$pbp7_{Go}$ $pPBP7_{Go}$ S56A	FC3598 strain complemented by expressing $pbp7_{Go}$ catalytically inactive mutant S56A allele from the plasmid pSEVA238 ($pPBP7_{Go}$ S56A). Kan^R.	This study
FC3601	Δ$pbp7_{Go}$ $pDacB_{Ec}$	FC3598 strain complemented by expressing $dacB_{Ec}$ WT allele from the plasmid pSEVA238 ($pDacB_{Ec}$). Kan^R.	This study
FC3602	Δ$pbp7_{Go}$ $pPbpG_{Ec}$	FC3598 strain complemented by expressing $pbpG_{Ec}$ WT allele from the plasmid pSEVA238 ($pPbpG_{Ec}$). Kan^R.	This study
FC3603	ldt_{Go}::ldt_{Go}*6his*	*G. oxydans* strain in which the ldt_{Go} allele has been replaced by ldt_{Go}-6Hi*s*.	This study

FC3632	pSEVA235	FC1604 strain carrying the pSEVA235 plasmid.	This study
FC3633	pSEVA235-Pldt$_{Go}$	FC1604 strain carrying the pSEVA235-Pldt$_{Go}$ plasmid.	This study
***Escherichia coli* strains:**			
FC3607	DH5alpha	*F^{-} φ80lacZΔM15 Δ(lacZYA-argF)U169 recA1 endA1 hsdR17(r$_{K}^{-}$, m$_{K}^{+}$) phoA supE44 λ^{-}thi-1 gyrA96 relA1*	2
FC3608	DH5alpha λ-PIR	*endA1 hsdR17 glnV44 (= supE44) thi-1 recA1 gyrA96 relA1 φ80dlacΔ(lacZ)M15 Δ(lacZYA-argF) U169 zdg-232::Tn10 uidA::pir+*	3
FC229	BL21	F^{-} *ompT hsdS$_{B}$* (r$_{B}^{-}$m$_{B}^{-}$) *gal dcm rne*131 (DE3)	ThermoFisher
FC2302	S17-1 λ-PIR	*TpR SmR recA, thi, pro, hsdR-M+RP4: 2-Tc:Mu: Km Tn7 λpir*	4
FC2050	BL21 pET28-LDT$_{Go}$	FC229 strain transformed with pET28-LDT$_{Go}$ plasmid. For peptidoglycan (PG) analysis and protein purification. Kan^{R}.	This study
FC2243	BL21 pET28-LDT$_{Go}$ C264A	FC229 strain transformed with pET28-LDT$_{Go}$ C264A plasmid. For PG analysis and protein purification. Kan^{R}.	This study
FC2246	BL21 pET28-LDT$_{Ap}$	FC229 strain transformed with pET28-LDT$_{Ap}$. For PG analysis. Kan^{R}.	This study
FC2247	BL21 pET28-LDT$_{Bcn}$	FC229 strain transformed with pET28-LDT$_{Bcn}$ plasmid. For PG analysis and protein purification. Kan^{R}.	This study
FC2240	BL21 pET28-LDT$_{Bcn}$ C354A	FC229 strain transformed with pET28-LDT$_{Bcn}$ C354A plasmid. For PG analysis and protein purification. Kan^{R}.	This study
FC3604	BL21 pET22-PelB-LDT$_{Go}$	FC229 strain transformed with pET22-PelB-LDT$_{Go}$ plasmid. For PG analysis and protein purification. Amp^{R}.	This study
***Vibrio cholerae* strains:**			
FC1110	N16961 *wild-type strain*	N16961, wild-type El Tor Clinical Isolate (Sm^{R})	5
FC3605	N16961 Δ*dacA1*	N16961 deletion mutant of the DD-CPase gene *dacA1*	6
**Other bacteria:**			
FC1606	*Acetobacter pasteurianus*	*Acetobacter pasteurianus* wild-type strain DSM2324	DSM collection
FC163	*Burkholderia dolosa*	*Burkholderia dolosa* wild-type strain AU0158	AU collection
FC760	*Burkholderia cepacia*	*Burkholderia cepacia* wild-type strain ATCC 25416	ATCC collection
FC3606	*Burkholderia multivorans*	*Burkholderia multivorans* wild-type strain DSM13243	DSM collection
FC178	*Burkholderia cenocepacia*	*Burkholderia cenocepacia* wild-type strain J2315	JCM collection

Supplementary Table 3. Plasmids.

FC code	Name	Description	Reference
FC3610	pKOS6b	Suicide vector for construction of deletion mutants in *G. oxydans*. Kan^R.	[7]
FC3611	pKOS6b-*gox2269*	pKOS6b derivative for clean deletion of *gox2269*. KanR.	This study
FC2095	pKOS6b-*gox1074*	pKOS6b derivative for clean deletion of *gox1074 (ldt_{Go})*. Kan^R.	This study
FC3612	pKOS6b-ldt_{Go}::ldt_{Go}*6his*	pKOS6b derivative for allelic exchange of the ldt_{Go} locus with a 6-His tagged version. Kan^R.	This study
FC3613	pKOS6b-*gox0607*	pKOS6b derivative for clean deletion of *gox0607* ($pbp7_{Go}$). Kan^R.	This study
FC3614	pKOS6b-ldt_{Go}::$ycbB_{Ec}$	pKOS6b derivative for allelic exchange of the ldt_{Go} locus with *ycbB* from *E. coli*. Kan^R.	This study
FC2626	pSEVA238	Plasmid used for complementation in *G. oxydans*. Expression of genes under the control of the xylS-Pm promoter. Activated by benzoate or m-toluate. Kan^R	[8]
FC3615	pGox1074 ($pLDT_{Go}$)	pSEVA238 derivative for expression of Gox1074 (LDT_{Go}). Kan^R.	This study
FC3616	pGox1074 H245A	pSEVA238 derivative for expression of Gox1074 (LDT_{Go}) H245A point mutant. Kan^R.	This study
FC3617	pGox1074 C264A	pSEVA238 derivative for expression of Gox1074 (LDT_{Go}) C264A point mutant. Kan^R.	This study
FC3618	$pLDT_{Go}$ ΔSP	pSEVA238 derivative for expression of LDT_{Go} ΔSP, carrying an N-terminal deletion of 30 residues. Kan^R.	This study
FC3619	$pLDT_{Go}$ SP_{YcbB}	pSEVA238 derivative for expression of LDT_{Go} SP_{YcbB}, in which the 52 first aa have been replaced by the SP (first 100 aa) of $YcbB_{Ec}$. Kan^R.	This study
FC3620	$pLDT_{Bcn}$	pSEVA238 derivative for expression of LDT_{Bcn} from *B. cenocepacia*. Kan^R.	This study
FC3621	$pPBP7_{Go}$	pSEVA238 derivative for expression of $PBP7_{Go}$. Kan^R.	This study
FC3622	$pPBP7_{Go}$ S56A	pSEVA238 derivative for expression of $PBP7_{Go}$ S56A point mutant. Kan^R.	This study
FC3623	$pDacB_{Ec}$	pSEVA238 derivative for expression of DacB from *E. coli*. Kan^R.	This study
FC3624	$pPbpG_{Ec}$	pSEVA238 derivative for expression of PbpG from *E. coli*. Kan^R.	This study
FC3625	pET28b(+)	Expression vector with T7 promoter and terminator flanking MCS, and optional C-terminal 6xHis-tag sequence. Kan^R.	Novagen

FC2037	pET28-LDT_{Go}	pET28b(+) derivative for inducible expression of LDT_{Go} from *G. oxydans* in *E. coli* Bl21. Kan^R.	This study
FC3626	pET28-LDT_{Go} C264A	pET28b(+) derivative for inducible expression of LDT_{Go} C264 point mutant from *G. oxydans* in *E. coli* Bl21. Kan^R.	This study
FC3627	pET28-LDT_{Ap}	pET28b(+) derivative for inducible expression of LDT_{Go} from *A. pasteurianus* in *E. coli* Bl21. Kan^R.	This study
FC3628	pET28-LDT_{Bcn}	pET28b(+) derivative for inducible expression of LDT_{Go} from *B. cenocepacia* in *E. coli* Kan^R.	This study
FC3629	pET28-LDT_{Bcn} C354A	pET28b(+) derivative for inducible expression of LDT_{Go} C354A point mutant from *B. cenocepacia* in *E. coli* Bl21. Kan^R.	This study
FC2025	pET28-*ldcA*	pET28b(+) derivative for purification of LdcA in *E. coli* Bl21. The protein carries a C-terminal 6-His tag. Kan^R.	[9]
FC1168	pET28-*ldtA*	pET28b(+) derivative for purification of LdtA from *V. cholerae* in *E. coli* Bl21. The protein has a N-terminal deletion of the signal peptide and carries a C-terminal 6-His tag. Kan^R.	[10]
FC2055	pET22b(+)	Expression vector with T7 promoter and terminator flanking MCS, *pelB* leader sequence for potential periplasmic localization and optional C-terminal 6xHis-tag sequence. Amp^R.	Novagen
FC3630	pET22-PelB-LDT_{Go}	pET22b(+) derivative for inducible expression and purification of PelB-LDT_{Go} *G. oxydans* in *E. coli* Bl21. The first 52 aa of LDT_{Go} are replaced by the PelB sequence for expression in the periplasmic space; the protein carries a C-terminal 6-His tag. Amp^R.	This study
FC2778	pET22-KP27	pET22b(+) derivative for purification of KP27 from *Klebsiella* phages. Amp^R.	[11]
FC2048	pSEVA235	*lacZ* transcription reporter plasmid. Kan^R.	[8]
FC3631	pSEVA235-$Pldt_{Go}$	pSEVA235 derivative for testing the ldt_{Go} promoter region (500 bp upstream of the *gox1073* gene). Kan^R.	This study

Supplementary Table 4. Primers for RT-PCR.

FCP code	Name	Sequence
FCP6599	gox1074_qPCR_fw	TTCTGACGCTTGCGAAGGAT
FCP6600	gox1074_qPCR_rv	ATATTTTCGCCCCGTCGTCC
FCP6601	gox0607_qPCR_fw	ATGACCAACACGACCTTCCG
FCP6602	gox0607_qPCR_rv	ATTGGGTCATGGTTCGGGAC
FCP6603	gox1522_qPCR_fw	CCTGAACCAGATCCGCATGA
FCP6604	gox1522_qPCR_rv	GGCGCCATCTTGTTCTTCAC

Supplementary Table 5. Identified muropeptides.

Muropeptide	Composition*	Monoisotopic mass (Da)	*m/z*			
			Theoretical	Charge	Observed	Difference
Monomers						
M1	NAG-NAM-A	569.243	570.251	1	570.252	0.001
M2 ($M2^{Glu}$)	NAG-NAM-A-E	698.286	699.294	1	699.308	0.014
$M2^{Gln}$	NAG-NAM-A-Q	697.302	698.3096	1	698.313	0.003
$M2^{Phe}$	NAG-NAM-A-F	716.312	717.3194	1	717.314	-0.005
$M2^{Trp}$	NAG-NAM-A-W	755.323	756.3303	1	756.330	0.000
M3	NAG-NAM-A-E-mDAP	870.371	871.3784	1	871.380	0.002
$M3^{Anh}$	NAG-NAM^{Anh}-A-E-mDAP	850.344	851.352	1	851.355	0.003
$M3^{NH_2}$	NAG-NAM-A-E-$mDAP^{NH_2}$	869.387	870.394	1	870.403	0.009
M4	NAG-NAM-A-E-mDAP-A	941.408	942.416	1	942.432	0.016
$M4^{Anh}$	NAG-NAM^{Anh}-A-E-mDAP	921.382	922.389	1	922.389	0.000
$M4^{Met}$	NAG-NAM-A-E-mDAP-M	1001.411	1002.419	1	1002.434	0.015
M5	NAG-NAM-A-E-mDAP-A-A	1012.445	1013.453	1	1013.456	0.003
Dimers						
$D33^{Anh}$	M3-$M3^{NH_2}$ ($mDAP^3$-$mDAP^3$, LD3,3-crosslink)	1702.704	852.360	2	852.364	0.004
$D43^{NH_2}$	M4-$M3^{NH_2}$ (A^4-$mDAP^3$, DD-crosslink)	1792.784	897.400	2	897.415	0.015
$D43^{NH_2\ Anh}$	$M4^{Anh}$-$M3^{NH_2}$ (A^4-$mDAP^3$, DD-crosslink)	1772.758	887.387	2	887.385	-0.002
D44	M4-M4 (A^4-$mDAP^3$, DD-crosslink)	1864.805	933.410	2	933.416	0.006
$D44^{Met}$	M4-$M4^{Met}$ (A^4-$mDAP^3$, DD-crosslink)	1924.808	963.412	2	963.411	-0.001
$D44^{Anh}$	M4-$M4^{Anh}$ (A^4-$mDAP^3$, DD-crosslink)	1844.779	923.397	2	923.402	0.005
D45	M4-M5 (A^4-$mDAP^3$, DD-crosslink)	1935.842	968.929	2	968.931	0.002
D13	M1-M3 (A^1-$mDAP^3$, LD1,3-crosslink)	1421.603	711.809	2	711.834	0.025
$D13^{NH_2}$	M1-$M3^{NH_2}$ (A^1-$mDAP^3$, LD1,3-crosslink)	1420.619	711.3174	2	711.316	-0.001
D14	M1-M4 (A^1-$mDAP^3$, LD1,3-crosslink)	1492.640	747.328	2	747.343	0.015
D15	M1-M5 (A^1-$mDAP^3$, LD1,3-crosslink)	1563.677	782.847	2	782.846	-0.001

Trimers						
T444	M4-M4-M4 (2x mDAP^3-mDAP^3, DD-crosslink)	2788.202	930.408	3	930.410	0.002
T144	M1-M4-M4 (A^1-mDAP^3, LD1,3-crosslink and A^4-mDAP^3, DD-crosslink)	2416.037	806.354	3	806.353	-0.001
T143^{NH_2}	M1-M4-M3^{NH_2} (A^1-mDAP^3, LD1,3-crosslink and A^4-mDAP^3, DD-crosslink)	2344.016	782.347	3	782.348	0.001
Endopeptidase products						
Tripeptide	E-mDAP-A	390.175	391.183	1	Not found	-
Tripeptide^{Met}	E-mDAP-M	450.178	451.186	1	451.189	0.003
M43	M4-tripeptide (A^4-mDAP^3, DD-crosslink)	1313.572	657.794	1	657.795	0.001
chain M1-M1	M1-M1 (β1→4 glycosidic bond)	1118.460	1119.468	1	1119.463	-0.005
chain M1-M1-M3	M1-M1-M3 (2x β1→4 glycosidic bond)	1968.805	985.4101	2	985.412	0.002
chain M1-M1-M4	M1-M1-M4 (2x β1→4 glycosidic bond)	2039.842	1020.929	2	1020.928	-0.001

*NAG: N-acetylglucosamine; NAM: N-acetylmuramic acid; ^{Anh}: anhydro N-acetylmuramic acid; A: alanine; E: glutamic acid (Glu); mDAP: meso-diaminopimelic acid. ^{NH_2}: amidated L-center of mDAP. Q: glutamine (Gln); F: phenylalanine (Phe); W: tryptophan (Trp); M: methionine (Met).

Supplementary Table 6. Setup for the molecular dynamics simulations.

	LDT_{Go}	LDT_{Go}-PG complex
Starting structure	X-ray / AF2	X-ray
Box dimensions ($Å^3$)	93 x 93 x 93	96 x 92 x 97
Minimum distance (Å) between any solute atom and the edge of the periodic box	15	12
Water molecules	24641	22538
Ions	20 K^+ / 15 Cl^-	19 K^+ / 9 Cl^-
Total number of atoms	78268	72521

SUPPLEMENTARY REFERENCES

1 Mason, L. M. C., G.W. Phenotypic Characteristics Correlated with Deoxyribonucleic Acid Sequence Similarities for Three Species of Gluconobacter: G. oxydans (Henneberg 1897) De Ley 1961, G. frateurii sp. nov., and G. asaii sp. nov. *International Journal of Systematic Bacteriology* **39**, 174-184 (1989).

2 Hanahan, D., Jessee, J. & Bloom, F. R. Plasmid transformation of Escherichia coli and other bacteria. *Methods Enzymol* **204**, 63-113 (1991). https://doi.org:10.1016/0076-6879(91)04006-a

3 Platt, R., Drescher, C., Park, S. K. & Phillips, G. J. Genetic system for reversible integration of DNA constructs and lacZ gene fusions into the Escherichia coli chromosome. *Plasmid* **43**, 12-23 (2000). https://doi.org:10.1006/plas.1999.1433

4 Simon, R., Priefer, U. & Pühler, A. A Broad Host Range Mobilization System for In Vivo Genetic Engineering: Transposon Mutagenesis in Gram Negative Bacteria. *Nat Biotechnol* **1**, 784–791 (1983). https://doi.org:https://doi.org/10.1038/nbt1183-784

5 Heidelberg, J. F. *et al.* DNA sequence of both chromosomes of the cholera pathogen Vibrio cholerae. *Nature* **406**, 477-483 (2000). https://doi.org:10.1038/35020000

6 Moll, A. *et al.* A D, D-carboxypeptidase is required for Vibrio cholerae halotolerance. *Environ Microbiol* **17**, 527-540 (2015). https://doi.org:10.1111/1462-2920.12779

7 Kostner, D., Peters, B., Mientus, M., Liebl, W. & Ehrenreich, A. Importance of codB for new codA-based markerless gene deletion in Gluconobacter strains. *Appl Microbiol Biotechnol* **97**, 8341-8349 (2013). https://doi.org:10.1007/s00253-013-5164-7

8 Silva-Rocha, R. *et al.* The Standard European Vector Architecture (SEVA): a coherent platform for the analysis and deployment of complex prokaryotic phenotypes. *Nucleic Acids Res* **41**, D666-675 (2013). https://doi.org:10.1093/nar/gks1119

9 Hernandez, S. B., Dorr, T., Waldor, M. K. & Cava, F. Modulation of Peptidoglycan Synthesis by Recycled Cell Wall Tetrapeptides. *Cell Rep* **31**, 107578 (2020). https://doi.org:10.1016/j.celrep.2020.107578

10 Cava, F., de Pedro, M. A., Lam, H., Davis, B. M. & Waldor, M. K. Distinct pathways for modification of the bacterial cell wall by non-canonical D-amino acids. *EMBO J* **30**, 3442-3453 (2011). https://doi.org:10.1038/emboj.2011.246

11 Maciejewska, B. *et al.* Klebsiella phages representing a novel clade of viruses with an unknown DNA modification and biotechnologically interesting enzymes. *Appl Microbiol Biotechnol* **101**, 673-684 (2017). https://doi.org:10.1007/s00253-016-7928-3
