# Supplementary material for: A distinctive family of L,D-transpeptidases catalyzing L-Ala-mDAP crosslinks in Alpha- and Betaproteobacteria
Source: Nat Commun. 2024 Feb 13;15:1343. doi: 10.1038/s41467-024-45620-5 (PMC10864386; doi:10.1038/s41467-024-45620-5)
Supplement: Supplementary file 3 — Description of Additional Supplementary Files [file 41467_2024_45620_MOESM3_ESM.pdf]

Description of Additional Supplementary Files:

Supplementary Data 1. Molecular dynamics simulations input and output files and generated AlphaFold2 models. LDTgo_structure_initial_AF2_model.pdb is the model used to solve the crystallographic phase problem during structure determination. The model was also used as input for molecular dynamics simulations. NatComm_model-PG_MDS.pdb is a coordinate file with 5 models of the final output of the molecular dynamics simulations. Generated AlphaFold2 models used in Supplementary Figure 12:
WP_011252635.1_Gluconobacter_oxydans_AF2.pdb,
WP_041249327.1_Gluconacetobacter_diazotrophicus_AF2.pdb,
WP_124305792.1_Acetobacter_pasteurianus_AF2.pdb,
WP_253736052.1_Granulibacter_bethesdensis_AF2.pdb,
WP_254845249.1_Desulfovibrio_sp._AF2.pdb and
B4EIM9_BURCJ_Burkholderia_cenocepacia_AF2.pdb.

Supplementary Movie 1. Molecular dynamics simulations of LDT_{Go}. The video comprises: (i) a rotating view of a rainbow-colored cartoon representation of the X-ray crystal structure, the dots highlighting the gap corresponding to the capping loop that is missing in the electron density map; (ii) a rotating view of our all-atom model of the full-length protein, first displaying all atoms and then only a rainbow-colored cartoon representation; (iii) a molecular dynamics trajectory spanning 350 ns (showing one snapshot every 5 ns) of the LDT_{Go} model that relates to the histogram plot of Supplementary Figure 13A, highlighting that motion is largely restricted to the capping loop (C atoms in grey) and parts of the N-terminal Pro-rich belt (C atoms in dark blue); (v) repositioning of the camera to display an alternative orientation (30 frames); (vi) a molecular dynamics trajectory spanning 240 ns (showing one snapshot every 5 ns) of the belt-truncated LDT_{Go} in complex with one peptidoglycan strand (C atoms in violet); and (vi) zoom-in on the active site of LDT_{Go} to show how the catalytic thiol of Cys264 is poised for nucleophilic attack to the carbonyl carbon of L-Ala^1 (C atoms in pink) of the donor muropeptide (C atoms in violet). The simulations on the apo form of LDT_{Go} were fully unrestrained whereas for the PG complex, (i) C1 and C4 atoms of N-acetylglucosamine and of N-acetylmuramic were restrained with a weak harmonic force constant of 2 kcal mol^{-1} $Å^{-2}$) to preserve the PG lattice architecture, and (ii) a harmonic force constant of 10 kcal mol^{-1} $Å^{-2}$ was employed to keep the Cys264(SG)–L-Ala(C=O) distance between 3.9 and 4.0 Å.
