# Supplementary material for: A distinctive family of L,D-transpeptidases catalyzing L-Ala-mDAP crosslinks in Alpha- and Betaproteobacteria
Source: Nat Commun. 2024 Feb 13;15:1343. doi: 10.1038/s41467-024-45620-5 (PMC10864386; doi:10.1038/s41467-024-45620-5)
Supplement: Supplementary file 6 — Reporting Summary [file 41467_2024_45620_MOESM6_ESM.pdf]

nature portfolio

Corresponding author(s): Felipe Cava

Last updated by author(s): Jan 23, 2024

Reporting Summary

Nature Portfolio wishes to improve the reproducibility of the work that we publish. This form provides structure for consistency and transparency in reporting. For further information on Nature Portfolio policies, see our Editorial Policies and the Editorial Policy Checklist.

Statistics

For all statistical analyses, confirm that the following items are present in the figure legend, table legend, main text, or Methods section.

n/a	Confirmed	
☐	☒	The exact sample size (*n*) for each experimental group/condition, given as a discrete number and unit of measurement
☐	☒	A statement on whether measurements were taken from distinct samples or whether the same sample was measured repeatedly
☐	☒	The statistical test(s) used AND whether they are one- or two-sided<br>*Only common tests should be described solely by name; describe more complex techniques in the Methods section.*
☒	☐	A description of all covariates tested
☒	☐	A description of any assumptions or corrections, such as tests of normality and adjustment for multiple comparisons
☐	☒	A full description of the statistical parameters including central tendency (e.g. means) or other basic estimates (e.g. regression coefficient) AND variation (e.g. standard deviation) or associated estimates of uncertainty (e.g. confidence intervals)
☐	☒	For null hypothesis testing, the test statistic (e.g. *F*, *t*, *r*) with confidence intervals, effect sizes, degrees of freedom and *P* value noted<br>*Give P values as exact values whenever suitable.*
☒	☐	For Bayesian analysis, information on the choice of priors and Markov chain Monte Carlo settings
☒	☐	For hierarchical and complex designs, identification of the appropriate level for tests and full reporting of outcomes
☒	☐	Estimates of effect sizes (e.g. Cohen's *d*, Pearson's *r*), indicating how they were calculated

Our web collection on statistics for biologists contains articles on many of the points above.

Software and code

Policy information about availability of computer code

Data collection	No custom code was generated for this study. Biotek Gen5 [v.08] was used to collect OD measurements for growth curves and beta-galactosidase assays. Waters Empower 3.6 and Waters UNIFI 1.8.1 were used for LC and LC-MS and MS/MS data acquisition, respectively. Fuji LAS-3000 Imaging System was used to collect western blot images. Zeiss Zen 2 Blue edition [v2.0.0.0] software was used for collection of microscopy data.
Data analysis	No custom code was generated for this study. Graphpad Prism 9.0 was used for graphing and analyzing most data. Microscopy images were analyzed with Fiji/ImageJ [v1.53] and MicrobeJ plugin. Phylogenetic analysis was carried out using PhyloT v2 and iTOL [v6]. Multisequence alignments were performed with Clustal Omega or T-COFFE Expresso. Sequence alignments were visualized with Jalview v2. ESPript was used for rendering sequence similarities and secondary structure information from aligned sequences. Signal peptide predictions were performed with SignalP 6.0 17. Sequence logos were generated in R v4.3 using the ggseqlogo package. ColabFold [v1.0] and [v1.5.2] and was used to build AlphaFold2 models. Phenix [1.21] and Phaser [2.8.3] were used to process the initial model. Coot [0.9.5] was used to build the model and the structures were refined using Refmac5 [v5.8.0267] and PHENIX refine [v1.13]. The pmemd.cuda and ccptraj modules of AMBER 22 (https://ambermd.org/) were used for the molecular dynamics simulations.

For manuscripts utilizing custom algorithms or software that are central to the research but not yet described in published literature, software must be made available to editors and reviewers. We strongly encourage code deposition in a community repository (e.g. GitHub). See the Nature Portfolio guidelines for submitting code & software for further information.

Data

Policy information about availability of data

All manuscripts must include a data availability statement. This statement should provide the following information, where applicable:

- Accession codes, unique identifiers, or web links for publicly available datasets
- A description of any restrictions on data availability
- For clinical datasets or third party data, please ensure that the statement adheres to our policy

The crystal structure data of LDTGo presented in this study is available in the PDB database under accession code 8QZG [https://doi.org/10.2210/pdb8QZG/pdb]. The AlphaFold2 models generated in this study are provided in the Supplementary Data 1 file. All other data in this study are included in the published article, its supplementary information and Source Data files, and are freely available without restriction from the corresponding author upon request.

Research involving human participants, their data, or biological material

Policy information about studies with human participants or human data. See also policy information about sex, gender (identity/presentation), and sexual orientation and race, ethnicity and racism.

Reporting on sex and gender	N/A
Reporting on race, ethnicity, or other socially relevant groupings	N/A
Population characteristics	N/A
Recruitment	N/A
Ethics oversight	N/A

Note that full information on the approval of the study protocol must also be provided in the manuscript.

Field-specific reporting

Please select the one below that is the best fit for your research. If you are not sure, read the appropriate sections before making your selection.

☒ Life sciences ☐ Behavioural & social sciences ☐ Ecological, evolutionary & environmental sciences

For a reference copy of the document with all sections, see nature.com/documents/nr-reporting-summary-flat.pdf

Life sciences study design

All studies must disclose on these points even when the disclosure is negative.

Sample size	The sample size was not predetermined or calculated by statistical methods. We have followed common standard practice in the molecular biology and microbiology fields, choosing the sample size based on literature (e.g., PMID: 31289173) and variability observed in previous experience in the laboratory. We have typically used at least three biological replicates to account for random variation. This provided sufficient statistical power to rule out differences due to inherent biological variation. Only for measurement of bacterial width and length we have chosen larger sample sizes, as indicated in the corresponding figure legend.
Data exclusions	No data was excluded from the study and analyses.
Replication	Experiments described in the manuscript were fully replicated, with three or more biological replicates. Where chromatograms or microscopy images are shown, these are representative of three biological replicates.
Randomization	No specific randomization processes were necessary as the experimental outcome does not depend on the order in which samples were analyzed in the experiments. Appropriate controls were used in all assays. In all experiments, control and experimental groups were done in isogenic strains.
Blinding	Blinding was not performed as all measurements in the study are quantitative at defined timepoints, and knowing the order or identity of a sample does not affect the results.

Reporting for specific materials, systems and methods

We require information from authors about some types of materials, experimental systems and methods used in many studies. Here, indicate whether each material, system or method listed is relevant to your study. If you are not sure if a list item applies to your research, read the appropriate section before selecting a response.

Materials & experimental systems

n/a	Involved in the study
☐	☒ Antibodies
☒	☐ Eukaryotic cell lines
☒	☐ Palaeontology and archaeology
☒	☐ Animals and other organisms
☒	☐ Clinical data
☒	☐ Dual use research of concern
☒	☐ Plants

Methods

n/a	Involved in the study
☒	☐ ChIP-seq
☒	☐ Flow cytometry
☒	☐ MRI-based neuroimaging

Antibodies

Antibodies used

Mouse anti-(H)5 antibody (Qiagen, ref: 34660, dilution 1:10000) and rabbit anti-mouse IgG – HRP-conjugated (Sigma, ref: A9044-2ML, dilution 1:30000) were used in Western blots.

Validation

The antibodies are commercially available and have been validated by the manufacturer.

Validation of the anti-His antibody can be found on the Qiagen website (https://www.qiagen.com/us/products/discovery-and-translational-research/protein-purification/tagged-protein-expression-purification-detection/anti-his-antibodies-bsa-free?catno=34660).
Certificates of analysis for the antibodies made by Qiagen can be found by lot number at https://www.qiagen.com/us/knowledge-and-support/product-and-technical-support/quality-and-safety-data/cofa-search.

Validation and certificates of analysis for the anti-mouse antibody can be found on the Sigma-Aldrich website (https://www.sigmaaldrich.com/SE/en/product/sigma/a9044?utm_source=google&utm_medium=cpc&utm_campaign=8939553830&utm_content=100673246340&gclid=Cj0KCQiAwbitBhDIARIsABfFYILSG6s78D9ebq_BY2jzee-i7iBhR_U4y0Ju9BuEboo4cValAmOqLXcaAjg5EALw_wcB).

Plants

Seed stocks

N/A

Novel plant genotypes

N/A

Authentication

N/A
